# The *ANKH ΔE490*Mutation in Calcium Pyrophosphate Dihydrate Crystal Deposition Disease (CPPDD) Affects Tissue Non-specific Alkaline Phosphatase (TNAP) Activities

**DOI:** 10.2174/1874312900802010023

**Published:** 2008-04-10

**Authors:** John Wang, Hing Wo Tsui, Frank Beier, Kenneth P.H. Pritzker, Robert D. Inman, Florence W.L. Tsui

**Affiliations:** 1 Genetics and Development Division, Toronto Western Research Institute, Canada; 2 Department of Physiology and Pharmacology, University of Western Ontario, Canada; 3 Pathology and Laboratory Medicine, Mount Sinai Hospital, Canada; 4 Department of Immunology, University of Toronto, Canada

## Abstract

ANKH (human homolog of progressive ankylosis) regulates inorganic pyrophosphate (PPi) transport. Dominant *ANKH* mutations were detected in at least five multiplex families with calcium pyrophosphate dihydrate crystal deposition disease (CPPPD). The objective of this study is to assess the functional consequences of one CPPDD-associated *ANKH* mutation (ΔE490) in chondrogenic ATDC5 cells. Stable ATDC5 transfectants bearing myc-tagged constructs of wild-type *ANKH*, mutant *ANKH (ΔE490)* and *neo *controls were generated. Upon ITS (insulin, transferrin and selenium) induction, expression of chondrocyte markers including alkaline phosphatase activity in the various transfectants was assessed. The *ANKH ΔE490-* transfectants had low alkaline phosphatase activities throughout ITS treatment due to lower TNAP protein expression and the presence of intracellular low-molecular-weight inhibitors. Our results suggest that the interplay of ANKH and TNAP activities is tightly regulated.

## INTRODUCTION

Inorganic pyrophosphate (PPi) and phosphate (Pi) affect mineralization of articular tissues and have a role in bone formation. It has long been known that Pi/PPi ratio is a crucial determinant of the type of crystal product formed (hydroxyapatite [HA] *vs* calcium pyrophosphate dihydrate [CPPD]) [1,2]. The recessive *ank (progressive ankylosis)* mice have a loss-of-function mutation in the *Ank* gene that codes for a regulator of PPi transport, and are characterized by pathologic calcium apatite crystal deposition and eventual bony ankylosis of the affected joints [3]. Patients with two rare diseases {craniometaphyseal dysplasia (CMD) [4, 5] and familial CPPDD [6-10]} have various dominant *ANKH* (human homolog of *Ank*) mutations. Though it is likely that CMD patients have dominant negative *ANKH* mutations, while familial CPPDD patients have gain-of-function *ANKH* mutations [11], the precise mechanisms whereby these *ANKH* mutations lead to disease pathogenesis are not entirely clear.

In familial CPPDD, most of the *ANKH* mutations were located at the 5’ end of the transcript, either in the 5’UTR or in codons for amino acid 5 or 48. One *ANKH* mutation (*ΔE490)* was initially identified in a patient with sporadic CPPDD. DNA analysis revealed that two unaffected family members of this patient had the same heterozygous mutation (*ΔE490)*[6]. Examinations of the functional consequences of these *ANKH* mutations have thus far been focused on the effect of these mutations on extracellular PPi levels and the results remain controversial [6, 9, 12]. Similar to an *Ank *wild-type transgene, a BAC transgene with the *M48T* mutation rescued the joint phenotype in the *Ank* null mice. However, unlike patients with the *ANKH M48T *mutation, no pathological CPPDD was found in any of these *Ank* null mice with the *Ank M48T* transgene [11]. It remains unclear whether this was due to the lack of an appropriate environment for CPPD crystal formation in mice.

In embryonic chick growth plate chondrocytes, Ank overexpression led to increased alkaline phosphatase (TNAP) expression and activity [13]. It is likely that the regulation of TNAP expression and activity by Ank ensures that sufficient TNAP activity is available at the outer membrane surface of growth plate chondrocytes to remove PPi, which is a potent inhibitor of mineralization [2, 13, 14]. In murine cementoblasts, Pi treatment upregulated *Ank, ENPP-1 *(ectonucleotide pyrophosphatase-1) and *PiT-1* (a Na^+^/Pi cotransporter) expression, while *TNAP* expression was downregulated, suggesting that Pi regulates genes involved in Pi/PPi homeostasis [15].

It is now clear that Ank/ANKH has function(s) additional to regulating PPi export. For example, we recently showed that ANKH promotes early erythroid differentiation [16]. In this study, we asked whether ANKH affects chondrocyte differentiation in ATDC5 cells and also assessed the functional consequence of overexpressing mutant *ANKH (ΔE4 90) *in ATDC5 cells.

## MATERIALS AND METHODS

### Cell culture and DNA transfection.

ATDC5 cells [17] were cultured in DMEM/F12 medium with 5% FBS. *ANKH *cDNAs**(*wt *and *ΔE490*) were subcloned into pCMV Tag 5A (Stratagene) in frame with the *c-*myc tag at the 3’end. All constructs were sequenced to ensure no DNA changes during the cloning process. ATDC5 cells were transfected with the various constructs using FUGENE 6 (Roche) and the transfectants were selected and maintained in G418. To avoid clonal bias, populations of stable transfectants (from two separate transfections) were used in all experiments. Differentiation of ATDC5 transfectants was induced in culture medium with 1% ITS (Sigma) and the cultures were fed with ITS containing medium every 2 days.

### Real-time RT-PCR.

RNAs were prepared using the Trizol method. Oligo-dT primers were used for reverse transcription. Primer sequences are summarized in Table **1**. Multiple dilutions of the RT mix were used for amplification to ensure linearity. *G3DPH* or *β-actin* expression was used for normalization. PCR reactions in triplicates were carried out for each sample using iQ SYBR mix (BioRad). Melt curves were done at the end of each PCR reaction. Relative quantification of gene expression was carried out using the 2^-ΔΔCt^ method [18], and each sample was compared to expression of *neo*-transfectants at D0. At least two different RNA preparations and four separate experiments were carried out for relative quantification of expression of each gene transcript. For each gene expression, statistical significance (p-value <0.05) among the transfectants was calculated using Two-Way Analysis of Variance (ANOVA) and pairwise multiple comparison procedures (Holm-Sidak method).

### Immunoprecipitation and immunoblotting.

Lysates of the transfectants were first immuno-precipitated with an anti-myc or a rabbit anti-Ank (raised against the last 20 aa of Ank and affinity purified) antibody. The immunoprecipitates were run on SDS-PAGE, and transferred to Immobilon-P. After blocking with 3% BSA (Sigma) in TBST (10mM Tris pH 8, 500mM NaCl, 0.1% Tween 20) for 1h, the blots were incubated with a goat anti-Ank antibody [19] for 45 min and then with horse radish peroxidase (HRP)-conjugated anti-goat antibody (Jackson) for 30 min. Specific signals were detected by chemiluminescence using Supersignal^® ^West Femto maximum sensitivity substrates (Pierce Biotech) and imaging (BioRad).

### Alkaline phosphatase activity assay.

Lysates (containing 1μg proteins) from each of the transfectants were incubated with 10mM p-NPP (Sigma-Aldrich) in a bicarbonate buffer (50mM Na_2_CO_3_/NaHCO_2_, pH 10.2, 1mM MgCl_2_, 10μM ZnCl_2_) at 37^o^C for either 2 or 5 min (at the linear part of the reaction). 0.2M NaOH was added to stop the reaction and OD was measured at 410nM. At least four experiments were carried out using different transfectant preparations.

## RESULTS

### Expression of endogenous Ank versus transfected ANKH transcripts and proteins.

**Fig. (1):**
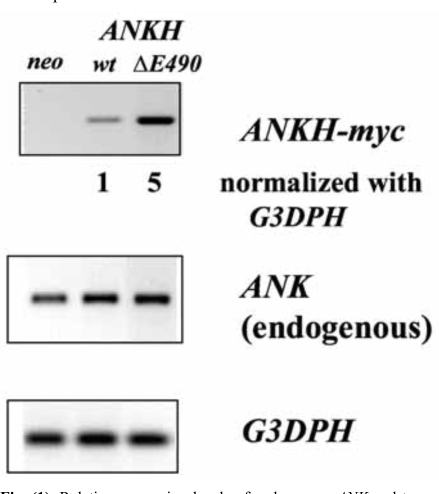
Relative expression levels of endogenous *ANK* and transfected *ANKH-myc* transcripts in various transfectants (*neo, wt* and *∆E490*). Expression of *G3DPH* was used for normalization.

To mimic the dominant mutations found in familial CPPDD cells, we transfected ATDC5 cells that express Ank proteins with *ANKH-myc* constructs with either a *wt* or a *ΔE490* mutation. The myc-tag enables us to differentiate the transfected from the endogenous transcript/protein. To assess the relative expression levels of various *ANKH-myc* transfectants, a primer pair with *ANKH* sequences for the forward primer and *myc* sequences for the reverse primer was used to carry out RT-PCR. After normalization to *G3DPH* expression, the relative expression of *ANKH ΔE490* was 5x that of *ANKH wt- *(Fig. **1**), while all transfectants including the *neo* controls expressed similar levels of endogenous *Ank* transcripts. As the primer efficiencies between these two primer-pairs might be different, the relative amount of transfected *vs* endogenous *ANKH/Ank* transcript in each transfectant is not known.

**Fig. (2):**
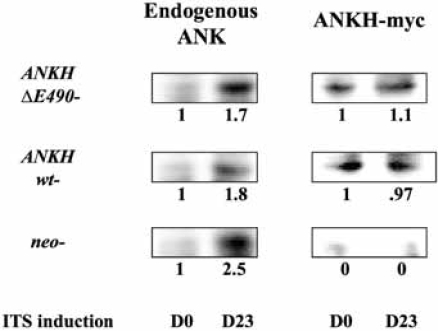
Relative expression levels of endogenous ANK versus ANKH-myc proteins in each transfectant in untreated (D0) and D23 of ITS induction.

We next assessed the relative level of expression of ANKH-myc *vs* Ank proteins in each transfectant. As we failed to obtain clean Western blots of the transfectants using anti-myc or anti-Ank antibodies, we first immunoprecipitated lysates of the transfectants using anti-myc antibody (to immuno-precipitate ANKH-myc proteins) or with a rabbit anti-Ank antibody (to immunoprecipitate all Ank/ANKH proteins) and probed the Western blots of the immunoprecipitates with a goat anti-Ank/ANKH antibodies. As the endogenous Ank protein is smaller than the ANKH-myc protein, both proteins can be differentiated on SDS-PAGE. As expected, the *neo* controls did not express any ANKH-myc proteins (Fig. **2**). For *ANKH wt-* transfectants, the relative levels of *ANKH-myc* transcript expression corresponded to the relative ANKH-myc protein expression (Fig. **2**, D0). Unexpectedly, *ANKH ΔE490-*transfectants only expressed one fifth of the predicted level of proteins. We also assessed whether ITS induction of ATDC5 led to an increase in endogenous Ank expression. On D23 of ITS induction, all transfectants showed approximately 2-fold increase in endogenous Ank proteins. As expected, there were no significant changes in the levels of ANKH-myc proteins over time in each transfectant (Fig. **2**, D23).

### Does ANKH play a role in chondrocyte differentiation?

**Fig. (3):**
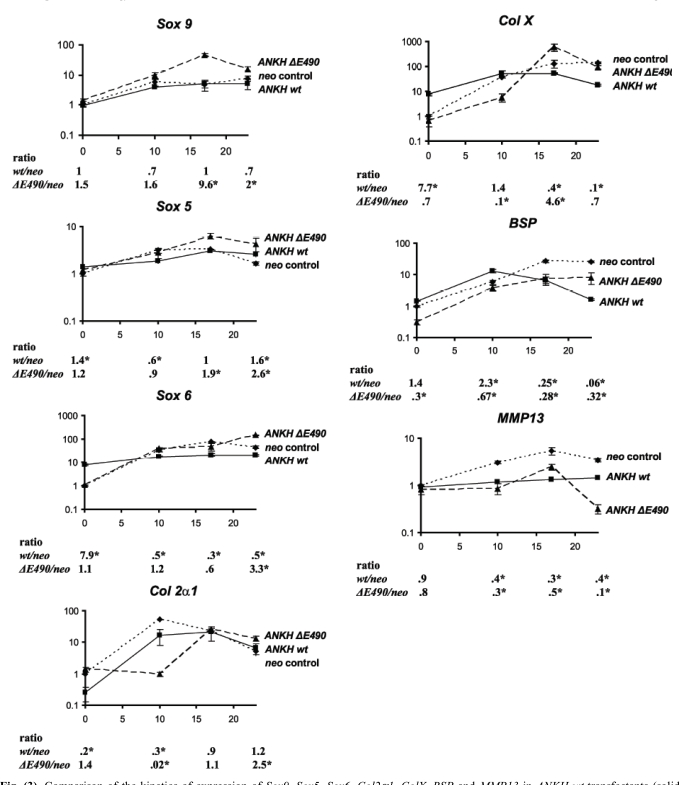
Comparison of the kinetics of expression of *Sox9, Sox5, Sox6, Col2α1, ColX, BSP* and *MMP13* in *ANKH wt*-transfectants (solid lines) *vs neo*-controls (dotted lines) *vs ANKH∆E490-* transfectants (dashed lines) at D0, D10, D17 and D23 of ITS treatment. Expression of *β-actin* was used for normalization. Ratio denotes expression in *ANKH*-transfectants (either *wt* or *∆E490*) over that of *neo*-controls. Arrow bars denote SD (n=4). * p-value<0.001 (Holm-Sidak method of pairwise multiple comparison).

*ANKH-*transfectants and the *neo*-controls were stimulated with ITS for 10, 17 and 23 days (ITS D10, D17, D23). At each time point, real-time RT-PCR was carried out to assess the relative expression of chondrocyte markers including three Sox transcription factors (*Sox 9, Sox 5*, and* Sox 6)*, one early chondrocyte marker (*collagen IIα1 [Col 2α1]*) and three hypertrophic chondrocyte markers (*bone sialoprotein [BSP], collagen X [ColX], and MMP-13*). As we found that *G3DPH* expression changes during the course of ITS induction of the transfectants, normalizations were carried out with *β-actin* expression instead. After normalization, the results were expressed as fold-changes relative to the expression in Day 0 *neo* transfectants. For each time-point, we also determined the ratio of expression (*ANKH-*transfectant: *neo* controls; listed below each plot at each time-point in Fig. **3**). For *neo*-transfectants, 10 days of ITS treatment resulted in a 6-, 3- and 34-fold increase in *Sox 9, Sox 5 *and* Sox 6 *expres-sion, respectively (Fig. **3**, *neo*). The high levels of expression of these three transcripts were maintained throughout the remaining course of ITS treatment (D17 and D23 ITS, Fig. **3**, *neo*). *Neo-*transfectants treated with ITS for 10 days had a 54-fold increase in *Col 2α1* expression. As expected, there was a 10-fold drop in *Col 2α1* expression when these transfectants were treated with ITS for 23 days. Maximal expression of all three hypertrophic chondrocyte markers (*Col X, BSP and MMP-13*) was detected in *neo*-transfectants treated with ITS for 17 and 23 days. Thus, the *neo*-transfectants showed all the expected patterns upon ITS treatments, suggesting that these cells behaved correctly. The same markers were used to assess whether over-expression of wild-type ANKH affects chondrogenesis and hypertrophic differentiation in ATDC5 *ANKH wt-*transfectants. Without ITS treatments, *ANKH wt-* transfectants had significantly higher expression of *Sox 6* and *Col X* (upregulated 7.9- and 7.7-fold respectively compared to that of *neo-*transfectants), suggesting that these transfectants were already more differentiated than the *neo*-controls. Interestingly, compared to the *neo-*controls, significantly lower expression of all three hypertrophic chondrocyte markers were detected in *ANKH wt-*transfectants treated with ITS for 17 and 23 days (Fig. **3**). It appears that the normal program of chondrocyte differentiation was disrupted by overexpression of wild-type ANKH.

### Overexpression of ANKH ΔE490- protein in ATDC5 cells changed the kinetics of the normal program of ITS-induced chondrocyte differentiation.

In view of two reports showing that *Col X* transcripts expression was induced in CH8/ATDC5 cells expressing the ANKH P5L- proteins [9, 12], we asked whether our stable transfectants with *ANKH ΔE490*- mutations had any effects on ITS induced ATDC5 differentiation, using the same set of seven different chondrocyte markers. Results from quantitative RT-PCR showed that in contrast to *ANKH wt-* transfectants, expression of six markers in the *ANKH ΔE490-*transfectants were similar to that of the *neo-*controls without ITS stimulation. There was a three-fold less *BSP* expression in the *ANKH ΔE490-*transfectants. However, compared to ITS treated *neo-* transfectants, the relative expression of *Sox 9* and *Sox 5* was significantly higher in D17 and D23 ITS stimulated *ANKH ΔE490-*transfectants, while that of *Sox 6* was significantly higher in D23 ITS treated *ANKH ΔE490-*transfectants (Fig. **3**). Compared to *neo-*transfectants, the mutant transfectants had a 4.6-fold higher levels of *Col X* expression with D17 ITS treatments, suggesting that there is a lag in the initial phase of chondrocyte differentiation in the *ANKH* mutant transfectants. *ANKH ΔE490*-transfectants significantly down- regulated *BSP* expression at D23 ITS, though the extent of the downregulated *BSP* expression was significantly lower than that of the *ANKH wt*- transfectants (p<0.001). Dysregulation of *MMP-13* expression was most evident in *ANKH ΔE490*-transfectants, especially at D23 ITS (10x downregulated compared to that of neo-controls; Fig. **3**). In summary, overexpression of ANKH wt proteins in ATDC5 cells promoted higher expression of *Sox 6* and *Col X* in the absence of ITS stimulation, and this appears to modulate the normal induction of chondrogenesis upon ITS treatment. In contrast, it appears that overexpression of ANKH ΔE490- proteins delayed the normal program of ITS-induced chondrogenesis in ATDC5 cells.

### Differential alkaline phosphatase (ALP) activities in *ANKH* transfectants.

**Fig. (4):**
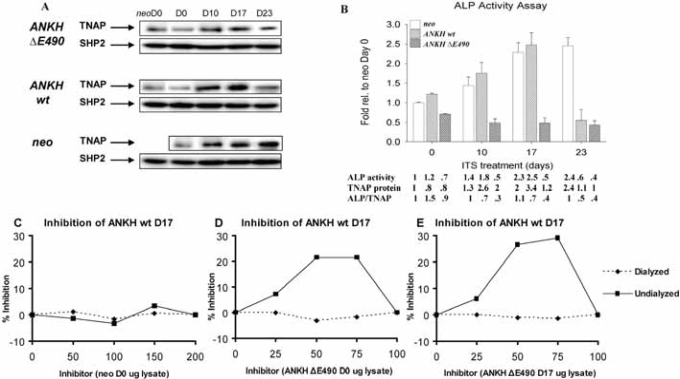
Alkaline phosphatase (ALP) activity in various transfectants. **(A)** Western blots showing TNAP protein expression. Quantification of relative TNAP expression was normalized with SHP-2 expression (also used as a specificity control). **(B)** Relative ALP activity of various transfectants at different time points (D0, D10, D17, and D23) of ITS treatment expressed as fold relative to that of *neo*-controls at D0. There is a statistical significant difference (Two-Way Analysis of Variance p-value <0.001) among the various transfectants and the *neo*-controls at each time point. Arrow bars denote SD (n=4). Relative ALP activity, TNAP protein levels (estimated by Western blot analysis and normal-ized with the expression of SHP-2), and ALP/TNAP ratio were tabulated. (C,D,E) Mixing experiments showing the presence of inhibitors in the lysates from *ANKH ∆E490*-transfectants. Lysates from *ANKH wt*- D17 ITS and *neo*- D0 **(C)**, *ANKH wt*- D17 ITS and *ANKH ∆E490*- D0 ITS **(D)**, and *ANKH wt*- D17 ITS and *ANKH ∆E490*- D17 ITS **(E)** were mixed in different ratios and assayed for ALP activity in the mixtures which had 1µ g of protein in all cases. The ALP activity from 1μg of individual transfectant lysate was also measured, and from this, the expected ALP activities in the different mixtures were estimated. Percentage (%) inhibition of each mixture was plotted against different amount of inhibitors (arbitrary numbers from 0 to 200). Solid lines showed % inhibition of undialyzed lysate mixtures and dotted lines showed % inhibition of dialyzed lysate mixtures.

It has been shown that ITS induction of ATDC5 cells resulted in an increase in ALP activities [17]. As expected, the *neo-*transfectants showed increased ALP activity that plateaued after D17 of ITS treatment. This increase in ALP activity was due to a correspondingly  increase in  TNAP proteins (normalized with the expression of SHP-2 protein; Fig. (**4A**,**4B**)). Similarly, *ANKH wt-* transfectants had comparable increase in ALP activity upon ITS treatment until D23, when the activity dropped below the D0 level (Fig. **4B**). The low level of ALP activity at D23 ITS was due to less TNAP protein expression as shown by Western blotting (normalized with the expression of SHP-2 protein; Fig. **4A**,**4B**). Unexpectedly, *ANKH ΔE490-*transfectants showed no increase in ALP activity throughout the course of ITS induction (Fig. **4B**). Compared to ITS treated *neo-* transfectants, *ANKH ΔE490-* D17 and D23 ITS transfectants showed about 2-fold less TNAP proteins; but the ALP activities were even lower than predicted by the protein levels (Fig. **4B**). We thus tested whether there were ALP inhibitors in the lysates from the *ANKH ΔE490-*transfectants by performing mixing experiments. Lysates from the *ANKH wt-* and *ΔE490-*transfectants were mixed in different ratios and assayed for ALP activity in the mixtures that had 1μg of protein in all cases. The ALP activity from 1μg of individual transfectant lysate was also measured, and from this, we estimated the expected ALP activities in the different mixtures. Mixing *ANKH wt-* with lysates from *neo-* transfectants resulted in the expected ALP activities in the mixtures (Fig. **4C**). However, mixing lysates from *ANKH ΔE490-* (either D0 or D17 ITS) resulted in inhibition of ALP activities in a dose-dependent manner, suggesting the presence of inhibitors in the *ANKH ΔE490-* lysates (Fig. **4D**,**4E**). The maximal inhibition observed was 30% (Fig. **4E**).

 Less inhibition (~21%) was observed when lysates from *ANKH ΔE490-* D0 transfectants were used as inhibitors (Fig. **4D**). These inhibitors were of low molecular weights as after dialysis of the lysates, the inhibitory activities were no longer present in these dialysed lysates. They were specifically found in *ANKH ΔE490-*transfectants as ATDC5 cells transfected with *ANKH* constructs bearing other known CPPDD-associated *ANKH* mutations did not have these inhibitors (data not shown). In summary, in *ANKH ΔE490-*transfectants, TNAP protein expression was less than that of *neo-*controls in D17 and D23 ITS, and intracellular low molecular weight inhibitors of ALP activities were present.

## DISCUSSION

In this study, we took advantage of the inducible ATDC5 cells to examine the effects of overexpressing *ANKH-*constructs (wild-type and mutant) on 1. pre-chondrocytes (D0 cells); 2. hypertrophic chondrocytes (ITS D23 cells); and 3. the kinetics of hypertrophic differentiation (D10/D17 cells represent the transition from pre- to hypertrophic chondrocytes). We showed that there are about 2-fold more endogenous Ank protein in ATDC5 cells treated with ITS for 23 days (Fig. **1B**). Over-expression of ANKH wt- proteins in ATDC5 cells at the pre-chondrocyte stage appears to disturb the normal induction of chondrogenesis by ITS. Over-expression of ANKH ΔE490- proteins appears to delay the kinetics of ITS induced chondrogenesis in ATDC5 cells. Thus, our results suggested that the levels of Ank in different stages of chondrogenesis are tightly regulated and its expression level possibly might even dictate the differentiation status of chondrocytes. In *ank/ank* mice which show a loss-of-function of Ank activities, embryonic and neonatal development of chondrocytes appear to be normal. This might argue against our results implicating that Ank plays a role in embryonic and neonatal chondrocyte development. However, one cannot rule out the possibility that some proteins (such as ENPP-1) might compensate for Ank function early in development. It remains unclear whether the altered kinetics of hypertrophic differentiation has any relevance to the development of CPPDD.

Our ATDC5 *ANKH ΔE490*-transfectants consistently expressed more *ANKH*-*myc* transcripts than ANKH-myc proteins. The reason for the discrepancy between mutant mRNA and protein expression is not entirely clear. It might be due to lability of the mutant protein. In contrast to our results, Williams’ group has recently shown that over-expression of wt Ank protein in ATDC5 cells had a 4.5-fold increase in *Col 10α1* expression at ITS D28 [11]. The reason(s) for this discrepancy is not clear, but could be due to the single time point chosen for analysis of *Col 10α1* expression. In situations where gain-of-function mutations are subjected to feedback transcriptional down-regulation, the outcome measured from cell culture systems will depend on the delicate balance between the gain-of function conferred by the mutation and the intrinsic**compensatory dampening of the response, leading to inconsistent and even contradictory results from different laboratories. We found that ITS stimulated the upregulation of endogenous ANK, but not the transfected ANKH (wt or mutant; Fig. **2**). Thus, the ratio of the endogenous *vs* transfected forms changed during the course of hypertrophic differentiation of the ATDC5 transfectants. This might lead to a milder phenotype than expected at ITS D17 and D23 in our results.

Our results, similar to others, also suggested that there is a tight relationship between Ank and TNAP. In hypertrophic transfectants, overexpression of ANKH-wt proteins led to a downregulation of ALP activity, presumably to maintain a balanced [Pi]/[PPi] ratio. In the *ANKH ΔE490-*transfectants, there was an abnormal down-regulation of TNAP expression throughout the course of ITS induction. The [Pi]/[PPi] ratio dictates the type of calcium crystals formed; hydroxyapatite (HA) crystals are formed when the ratiois >100 and CPPD crystal formation are promoted when the ratio is <3 [1]. Alkaline phosphatase is a key player in CPPDD, as the presence of TNAP in articular cartilage facilitates both formation and dissolution of CPPD crystals [20]. In hypertrophic ATDC5 cells, over-expression of ANKH wt- proteins led to a decrease in TNAP protein (2-fold less than *neo*-controls at D23 ITS) and ALP activity (4-fold less than *neo-*controls at D23 ITS). If the TNAP level in the extracellular matrix (ECM) is limiting, this could lead to less PPi being degraded by TNAP, favoring the formation of CPPD crystals. For the *ANKH ΔE490-*transfectants, we showed that not only less TNAP proteins than in *neo*-controls were detected, but also that dialyzable low-molecular-weight inhibitors of ALP were present. The identity of the inhibitor(s) has not been defined, though our preliminary results indicated that the inhibitor was not Pi [21]. We recently showed that endogenous inhibitors such as cysteine might interfere with TNAP’s ability to regulate CPPD crystal formation and dissolution [22], though it is not clear whether cysteine is the inhibitor in the *ANKH (E490-* transfectants.

It is likely that the biochemical heterogeneity observed in our study might explain the clinical heterogeneity in the familial CPPDD caused by the different *ANKH* mutations. The British patient, initially thought to have spontaneous CPP DD, has the *ANKH (E490* mutation. CPPDD in this patient was late-onset, polyarticular with structural arthropathy of both knees. Two other family members also have this heterozygous mutation, but with no clinical symptoms of CPP DD. However, one of them had bilateral total knee replacement surgery for “osteoarthritis” [6], but the other was normal at the age of 42. It is possible that other contributing factor(s) might be required in addition to the *ANKH (E490* mutation, before CPPDD will develop. Similar to the finding in ANK deficient mice bearing an *ANKH M48T *transgene [11],**results from our studies using the ATDC5 transfectants also did not show any evidence that the *ANKH (E490* mutation is an activating mutation.

Based on our results in this study, we hypothesize that CPPDD-associated ANKH proteins lead to dysregulation of modulators of Pi/PPi homeostasis such as TNAP. As Pi/PPi homeostasis is a dynamic process and probably subject to feedback mechanisms, it would be very difficult to replicate pathological [Pi]/[PPi] conditions in *in vitro* culture systems, leading to the generation of inconsistent and contradictory results. The long-time observation that both HA and CPPD crystals were found in CPPDD patients has been puzzling. It is likely that in these patients, the dynamic interplay of ANKH and TNAP led to the fluctuating local [Pi]/[PPi] ratio, resulting in the formation in the joints of either HA (when e[Pi]/e[PPi] ratio is >100) or CPPD crystals (when e[Pi]/e[PPi] is <3). Flare-ups of CPPDD probably occur in situations where the negative feedback loop is not optimal.

*ANKH* mutations have only been detected in rare families with CPPDD and one *ANKH *SNP was associated with sporadic cases in UK [9]. In one CPPDD family from New England, the disease was linked to a locus on chromosome 8q instead of chromosome 5p where ANKH is mapped [23]. A recent study suggested that ENPP-1 and TNAP are not major determinants of sporadic CPPDD susceptibility [24]. *ANKH* transcripts were upregulated in cartilage from four CPPDD patients [25]. Thus, one cannot rule out that dysregulation of ANKH and TNAP by factors such as cytokines might be important in the manifestation of sporadic CPPDD.

## CONCLUSIONS

In summary, we propose the following model for the development of CPPDD: under normal conditions, the interplay of ANKH and TNAP facilitates normal mineralization. Dysregulation of ANKH (either by mutation or induced by cytokines) lead to a dysregulation of TNAP. Together with other yet-to-be-identified factors, pathological mineralization ensued, leading to CPPDD disease expression.

## Figures and Tables

**Table 1.** RT-PCR Primer Sequences

Target	Primer Sequences (5’ to 3’)
*Sox9*	F: ATCGGTGAACTGAGCAGC GAC R: GCCTGCTGCTTCGACATCCA
*Sox5*	F: CTCGCTGGAAGCTATGACC R: GATGGGGATCTGTGCTTGTT
*Sox6*	F: GGATTGGGGAGTACAAGCAA R: CATCTGAGGTGATGGTGTGG
*Col2α1*	F: TTGAGACAGCACGACGTGGAG R: AGCCAGGTTGCCATCGCCATA
*Col X*	F: AAAGCTTACCCAGCAGTAGG R: ACGTACTCAGAGGAGTAGAG
*BSP*	F: GCATGCCTACTTTTATCCTC R: GTTCTCGTTGTCATAGACTTC
*MMP-13*	F:CATTCAGCTATCCTGGCCACCTTC R:CATCCACATGGTTGGGAAGTTCTG
*b-actin*	F: GTGGGCCGCTCTAGGCACCAA R: CTCTTTGATGTCACGCACGATTTC
*GADPH*	F: GCTGGCATTGCTCTCAATGA R: AGGCCCCTCCTGTTATTATG

**Table 2.** Significant Changes in Markers Expressed in Various ANKH-Transfectants Relative to that of the neo-Controls at Each Time-Point

Transfectant	ITS	*Sox9*	*Sox5*	*Sox6*	*Col2α1*	*ColX*	*BSP*	*MMP-13*	ALP Activity	TNAP Protein
*ANKH-wt*	D0		↑1.4x	↑7.9x	↓5x	↑7.7x				
	D10		↓1.7x	↓2x	↓33x		↓10x	↓2.5x		
	D17			↓33x		↓2.5x		↓3.3x		
	D23		↑1.6x	↓2x		↓10x	↓33x	↓2.5x	↓4x	↓2x
										
*ANKH- ΔE490*	D0						↓3.3x			
	D10				↓50x	↓10x	↓2.5x	↓3.3x	↓2.8x	
	D17	↑9.6x	↑1.9x			↑4.6x	↓3.3x		↓5x	↓2x
	D23	↑2x	↑2.6x	↑3.3x	↑2.5x		↓10x	↓2.5x	↓6x	↓2x

## ABBREVIATIONS

ALP: Alkaline phosphatase
ANK: Progressive ankylosis
ANKH: Human homolog of progressive ankylosis
ANOVA: Two-way analysis of variance
BAC: Bacterial artificial chromosome
BSP: Bone sialoprotein
BSA: Bovine serum albumin
CMD: Craniometaphyseal dysplasia
Col2α1: Collagen II α1
Colx: Collagen X
CPPDD: Calcium pyrophosphate dihydrate crystal deposition disease
ECM: Extracellular matrix
ENPP-1: Ectonucleotide pyrophosphatase-1
FBS: Fetal bovine serum
MMP13: Matrix metalloproteinase 13
ITS: Insulin, transferring, selenium
TNAP: Tissue non-specific alkaline phosphatase
Pi: Inorganic phosphate
p-NPP: Para-nitrophenyl phosphate
PPi: Inorganic pyrophosphate
UTR: Untranslated region
